# The effects of cataract surgery on autonomic heart rate control: a prospective cross-sectional and analytical study

**DOI:** 10.6061/clinics/2019/e809

**Published:** 2019-09-04

**Authors:** Ricardo H Aoki, Italla Maria Pinheiro Bezerra, Alvaro Dantas de Almeida-Júnior, Renata Thaís de A. Barbosa, Vitor E Valenti, Fernando R Oliveira, Adriano L Roque, Hugo Macedo Ferraz e Souza Júnior, David M Garner, Rodrigo D Raimundo, Luiz Carlos de Abreu

**Affiliations:** I Laboratorio de Delineamento de Estudos e Escrita Cientifica, Centro Universitario Saude ABC, Santo Andre, SP, BR; II Programa de Mestrado em Politicas Publicas e Desenvolvimento Local da Escola Superior de Ciencias da Santa Casa de Misericordia, Vitoria, ES, BR; III Programa de Mestrado em Ciencias da Saude da Amazonia da Universidade Federal do Acre, Bolsista CAPES Brasil, Rio Branco, AC, BR; IV Centro de Estudos do Sistema Nervoso Autonomo, Faculdade de Filosofia e Ciencias, Universidade Estadual Paulista, Marilia, SP, BR; V Programa de Pos-Graduacao em Epidemiologia, Faculdade de Saude Publica, Universidade de Sao Paulo, Sao Paulo, SP, BR; VI Programa de Pos-Graduacao em Cardiologia, Universidade Federal de Sao Paulo, Escola Paulista de Medicina, Sao Paulo, SP, BR; VII Cardiorespiratory Research Group, Department of Biological and Medical Sciences, School of Health and Life Sciences, Oxford Brookes University, Headington Campus, Gipsy Lane, Oxford OX3 0BP, United Kingdom; VIII Graduate Entry Medical School, University of Limerick, Limerick, V94 T9PX, Ireland

**Keywords:** Autonomic Nervous System, Heart Rate Variability, Cardiovascular System, Cataract, Ophthalmologic Surgical Procedures

## Abstract

**OBJECTIVES:**

We aimed to evaluate the effects of cataract surgery on cardiac autonomic modulation.

**METHODS:**

A cross-sectional and analytical study was conducted at the Hospital Maria Braido in the city of São Caetano do Sul, São Paulo, between 2015 and 2016. We investigated 19 patients of both sexes who were all over 50 years old; all patients had a diagnosis of senile or bilateral cataracts and were recommended to undergo implantation of the intraocular lens. Heart rate variability (HRV) was evaluated before, during and after cataract surgery.

**RESULTS:**

There were no significant changes in the time and geometric domains of HRV before, during or after surgery. The high-frequency (HF) band in normalized units (nu) on the spectral analysis significantly increased (*p*=0.02, Cohen's *d*=0.9, large effect size). However, the low-frequency (LF) band in nu significantly decreased during surgery (*p*=0.02, Cohen's *d*=0.9, large effect size).

**CONCLUSION:**

Throughout the intraocular lens implantation cataract surgery, there was an increase in parasympathetic modulation and a decrease in the sympathetic component of the heart rate (HR). We propose that this result is attributable to the supine position of the patients during surgery and the trigeminal reflex.

## INTRODUCTION

Visual deficit is present in approximately 285 million people worldwide; 39 million are considered blind and 246 million have imperfect vision. Cataracts are the major cause of treatable blindness in developing countries and account for 33% of the worldwide causes of visual impairment; cataracts are often diagnosed by lens opaqueness 1.

Cataracts are an ocular disease that progressively affects visual quality during the natural aging process through loss of lens transparency 2,3. The loss of visual acuity prompts a reduction in the quality of life of elderly people worldwide 4,5; cataracts also result in an economic burden on the patient's family 6, deteriorating global health and increased mortality 7,8.

Risk factors such as progressive aging, type 2 diabetes, prolonged exposure to ultra-violet (UV) sunlight, tobacco use, alcohol consumption, and myopia heighten the probability of developing cataracts. The diagnosis typically occurs through a combination of individual complaints from the patient and/or unbiased signs on an ophthalmologic examination 9.

The surgical treatment of cataracts restores the transparency of the lens and amends defects prior to the refractive cataract 10. Surgical intervention is an effective treatment for cataract correction that involves lens removal and replacement of the lens with a suitable artificial lens. Cataract surgery has become one of the easiest and most efficient procedures of all modern outpatient procedures 11.

Nevertheless, ophthalmic surgery is suspected to induce deviations in the autonomic nervous system, as in any type of surgery 12,13. One way to evaluate the autonomic nervous system is through heart rate variability (HRV), which provides physiological information regarding cardiac autonomic regulation. HRV indicates the influence of the parasympathetic and sympathetic autonomic nervous system on the heart rhythm 14.

A vital feature to consider during ophthalmic surgery is the oculovagal reflex, which is mediated through the afferent neurons of the trigeminal nerve (ophthalmic division) and the vagus nerve. Vagal efferent nerves innervating the heart reduce sinoatrial node output, leading to hypotension and bradycardia. The potentially damaging side-effect of this reflex is that it could increase the chance of cardiac arrest 15,16.

Previously, it has been demonstrated that there is an association between ophthalmic surgery and the autonomic cardiac regulation system. Keyl et al. 17 evaluated HRV in healthy patients under local or general anesthesia for cataract surgery. The authors reported that general anesthesia had no significant negative impact on HRV compared to local anesthesia. The changes in heart rate (HR) autonomic control during cataract surgery were not fully elucidated; however, their key research focus was the influence of anesthesia on HRV.

In 2009, Cobb et al. 18 performed a retrospective study with pediatric and young adults undergoing surgery for a fracture of the orbital floor or medial wall. It was shown that the absence or presence of the oculovagal reflex was not considered by the medical team and the trend for autonomic alterations was more distinguished in the younger individuals.

Taken together, the aforementioned studies 15-18 provide substantial evidence that ophthalmic surgery is able to induce changes in cardiac autonomic modulation. Nevertheless, it is unclear whether cataract surgery has a substantial impact on HRV. Cardiac autonomic control during cataract surgery is an important issue that should be investigated to verify any cardiac risks during the ophthalmologic procedure. Consequently, the aim of this study was to evaluate HR autonomic control fluctuations induced by cataract surgery.

## METHODS

### Study design and population

This was a cross-sectional and analytical study including patients from the Hospital Maria Braido from São Caetano do Sul (São Paulo) who were diagnosed with cataracts and underwent suitable surgery via intraocular lens implantation between 2015 and 2016. All subjects provided their confidential and informed consent for participation in the research study. All study procedures were approved by the Ethics Committee in Research of the Faculty of Juazeiro do Norte and were in accordance with the 466/2012 National Health Resolution.

### Inclusion criteria

Adults over 50 years old of both sexes were included in this study. This specific age group was selected because the majority of patients in our institution are older than 50 years old. All patients were diagnosed with unilateral or bilateral senile cataracts (crystalline and idiopathic opacity) with a reduction in of visual acuity and adequate refraction.

### Exclusion criteria

Patients who had untreated or unrestrained cardiopulmonary, neurological, and other conditions that could prevent patient consent to the procedures and patients who underwent sedation were excluded. Accordingly, all included subjects received local anesthesia, were submitted to identical surgical procedures and did not receive any additional sedative medications. We also excluded subjects with traumatic cataracts, congenital cataracts, primary glaucoma, proliferative diabetic retinopathy, and conjunctivitis and patients who were administered other pharmacological treatments that influenced the autonomic nervous system (i.e., adrenergic and muscarinic blockers and agonists). Patients with morbidities such as type II diabetes, hypertension, sepsis, erysipelas, and functional heart disease were excluded.

### Experimental protocol

The clinical data of subjects, including their weight, age, height and body mass index (BMI), were collected. Data collection was performed in a room with the relative humidity between 50% and 60% and temperature between 20°C and 25°C. Subjects were recommended to not ingest caffeine, alcohol or other autonomic nervous system stimulants for 24 hours before the evaluation, and they remained at rest and avoided conversation during the data collection procedures. All procedures required for the data collection were discussed individually, and data were collected in the morning between 8:00 and 12:00 for circadian rhythm standardization.

The subjects refrained from taking medications for 24 hours prior to the experiments.

**Figure 1:**
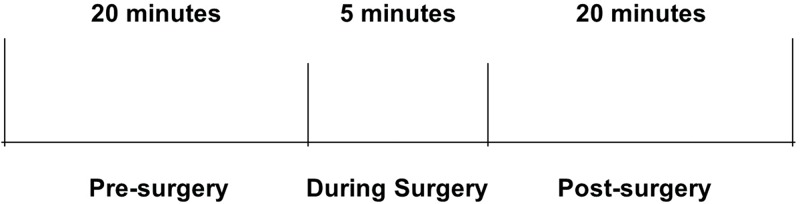
Experimental protocol.

During the protocol, the subjects remained seated and performed spontaneous breathing during the entire experimental protocol. After the resting (or control) period, the subjects were taken to the operating theater for cataract surgery, and the HR signal was recorded for five minutes. After completion of surgery, the subjects were returned to the same room where the postoperative data were recorded. The subjects were advised to remain seated, at rest, and to perform spontaneous breathing for 20 minutes (Figure 1).

All events required for data collection were explained individually, and subjects were instructed to remain at rest and avoid conversation and/or sudden movements during collection that could impact cardiac autonomic modulation.

### Surgical procedures

Unilateral cataract (independent of the diagnosis of the other eye) surgeries with intraocular lens implantation were completed. At the outset, topical lidocaine-based anesthesia was administered to the exposed part of the eyeball. This procedure was completed before the patient was transferred to the surgical center, with the subject maintaining a supine position on the surgical table. Asepsis was achieved through local antisepsis measures, and the blepharostat (an instrument for holding the eyelids apart) was suitably positioned in the sterile field.

The key incision, which was of 2.75 mm clear corneal biplanar incision on the lamina with an accessory incision (side port), was performed. The anterior chamber was then filled with trypan blue dye followed by 0.5% heavy intravenous bupivacaine diluted intramuscularly by the viscoelastic exchange of all these substances. Subsequently, the anterior capsule of the lens (capsulorhexis) was released and removed with an ultrahose. Then, hydrodissection and hydrodelineation were finalized with balanced saline solution (BSS) using a 5 ml syringe with a cannula. Emulsification of the nucleus was performed by the phaco-chop technique, followed by irrigation and aspiration of the cortex. Next, the anterior chamber was filled with viscoelastic material for implantation of the collapsible lens into the capsular bag, which held the cloudy natural lens (or cataract). Then, carbachol was applied to perform intraoperative miosis (excessive constriction of the pupil of the eye), and mechanical aspiration of all anterior chamber material was completed. To finish the procedure, the anterior chamber was filled with BSS to hydrate the incisions, a topical antibiotic was applied and occlusion of the operated eye was accomplished.

The surgical procedure took between 18 and 25 minutes to complete.

### HRV analysis

HRV was investigated following directives from the task force guidelines 14. Details of HRV analysis have been previously described 19. Time and frequency domain analyses were composed of standard deviations of normal-to-normal RR intervals (SDNN), the percentage of adjacent RR intervals with a duration difference greater than 50 ms (pNN50), root-mean square of differences between adjacent normal RR intervals at a specific time interval (RMSSD), LF (ranging between 0.04 Hz and 0.15 Hz) and HF (ranging from 0.15 Hz to 0.4 Hz) band comparisons, and the LF/HF ratio. Geometric indices of HRV were likewise evaluated with the triangular interpolation of RR intervals (TINN) and Poincaré plots (SD1, SD2 and SD1/SD2 ratio) 19,20.

To calculate the linear indices, we used HRV analysis software (Kubios^®^ HRV v.1.1 for Windows, Biomedical Signal Analysis Group, Department of Applied Physics, University of Kuopio, Finland) 21.

HRV was examined in the preoperative, intraoperative and postoperative periods.

### Statistical analyses

The sample size was calculated via online software from the website www.lee.dante.br, considering the resting RMSSD index for the correlation analysis. The level of statistically significant differences assumed was 40 ms, with a standard deviation of 20 ms, a risk of 0.1% alpha and a beta of 95%. The proposed sample size calculation recommended a minimum of 15 subjects.

The normality of the data was estimated by the Shapiro-Wilk test (z value >1.0). We considered a significant difference at the level of *p*<0.05 (or <5%).

To verify the significant difference between factors, we calculated the effect size with Cohen's *d*. We considered a large effect for values ≥0.9, a medium effect for values between 0.9 and 0.5 and a small effect for values between 0.5 and 0.25 22.

To evaluate the associations between resting HRV, age and BMI, we applied the Pearson correlation test for parametric distributions and the Spearman correlation test for nonparametric distributions. We assumed a strong correlation if the r coefficient >0.75 and a moderate correlation for an r coefficient between 0.5 and 0.3.

## RESULTS

We assessed 19 patients (42% female). Regarding comorbidities, we found that seven patients had one associated disease, while the others had more than one (Table 1).

Additional medications taken by subjects were only analgesics due to lumbar hernia.

Table 2 presents the age and BMI of the patients.

We observed no significant correlation between resting HRV and age or BMI (Table 3).

**Figure 2:**
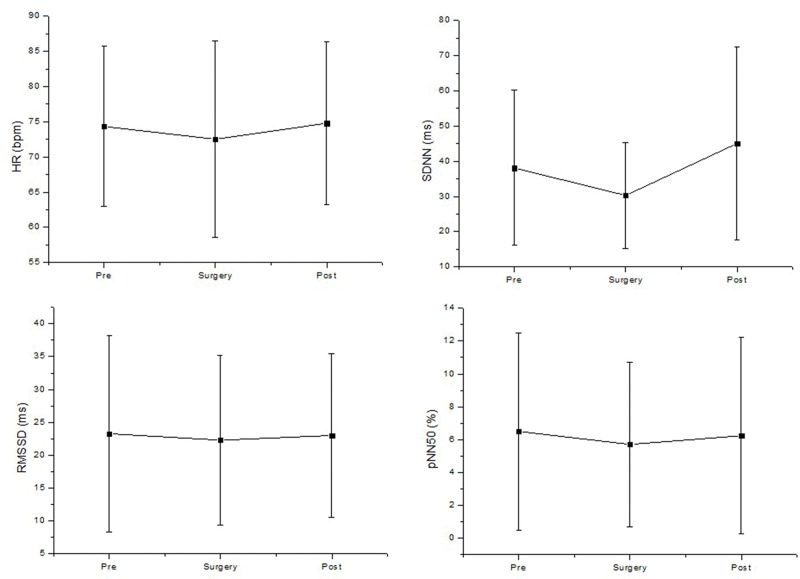
Time domain analysis of HRV before (pre), during surgery (surgery) and after surgery (post). pNN50: the percentage of adjacent RR intervals with a difference of duration greater than 50 ms; RMSSD: root-mean square of differences between adjacent normal RR intervals in a time interval; ms: milliseconds; SDNN: average standard deviation of normal RR intervals; HR: heart rate; ms: milliseconds.

The time domain analyses of HRV and HR are presented in Figure 2. We were unable to observe any significant deviations of the SDNN, RMSSD, pNN50 value and HR induced by cataract surgery.

**Figure 3:**
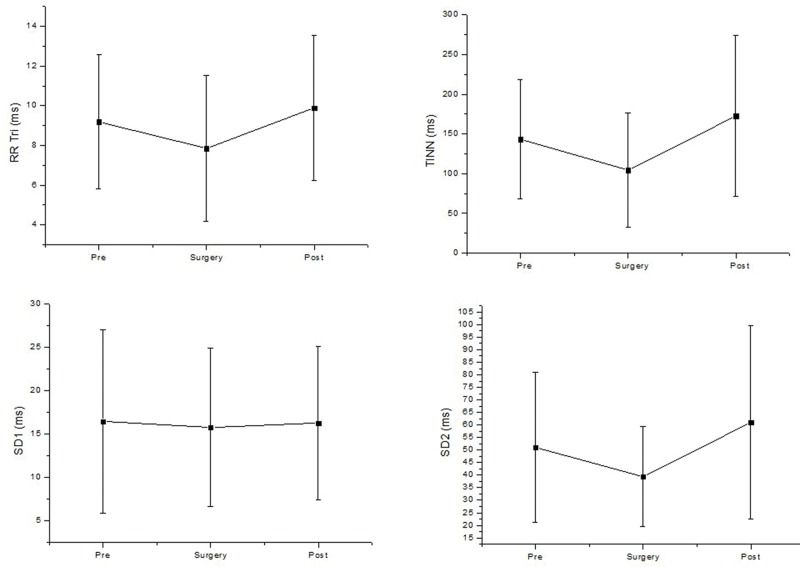
Geometric analysis of HRV before (pre), during surgery (surgery) and after surgery (post). SD1: standard deviation of the instantaneous variability of the beat-to beat HR; SD2: standard deviation of long-term continuous RR interval variability; TINN: triangular interpolation of the RR interval histogram; RRtri: triangular index. ms: milliseconds.

There was no deviation in the geometric analysis of HRV during surgery. No significant changes were detected for the TINN, triangular index (RRtri), and the SD1 and SD2 indices (Figure 3).

**Figure 4:**
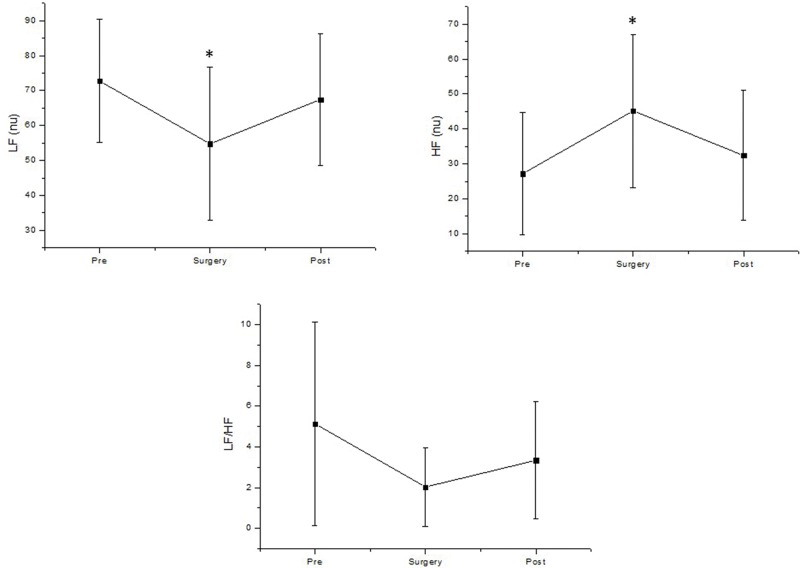
Frequency domain analysis of HRV before (pre), during surgery (surgery) and after surgery (post). LF: low frequency; HF: high frequency; LF/HF: low frequency/high frequency ratio; nu: normalized units. *p=0.02; Pre *vs* Surgery.

Equally, spectral analysis of HRV revealed that the parasympathetic regulation of HR increased during cataract surgery compared to control values before surgery through evaluation of the HF (nu) band (Cohen's *d*=0.9, large effect size); the LF band was significantly decreased during surgery (Cohen's *d*=0.9, large effect size) (Figure 4).

## DISCUSSION

Our study was completed to evaluate the acute effects of cataract surgery on the autonomic control of HR. We observed no change in the HR. However, HRV analysis indicated increased vagal regulation during the surgery. The involvement of the parasympathetic nervous system was demonstrated by the increase in the HF band during surgery compared to the HF bands before and after the surgical procedures.

Previous studies have investigated the autonomic nervous system in endodontic 23, spinal 24 and major abdominal surgeries 25. No previous study has assessed the autonomic nervous system in solely ophthalmologic or cataract surgeries.

We consider these results important for two reasons. The first is the increase in the vagal control of the HR due to the supine position 26. We advise that this is the principal influence on HRV during cataract surgery. All cataract surgeries are performed with the patients in the supine position.

The second reason is the stimulation of the ophthalmic supraorbital branch of the trigeminal nerve, inducing the trigeminal reflex 27. This trigeminal blink reflex induces bradycardia and even cardiac arrest during the intraoperative period. This reflex is strengthened by the stimulation of the orbicularis oculi muscles. The ciliary nerves refer input to the ciliary ganglion, which sends signals to the Gasserian ganglion and terminates in the body of the trigeminal ganglion. Brainstem zones activate the vagus nerve that sends efferent information, triggering a decrease in HR 28.

The complete pathway of the trigeminal blink reflex has not been fully elucidated, and there is little evidence in the scientific research literature. A previous study analyzed the brainstem reflex through a voxel-based morphometry (VBM) model of the human brainstem used to normalize functional magnetic resonance imaging 29. The authors highlighted important areas involved in this reflex, including the neurons involved in extending the lateral reticular formation at the pontine and medullar levels.

In this way, it is necessary to highlight that the ophthalmic branch of the trigeminal nerve is an afferent pathway, and as such, its impulses permeate the reticular formation and subsequently track the visceral nuclei of the vagus nerve 29,30.

In ophthalmologic surgery, cardiac autonomic modulation tends to stimulate the vagal nerve because of ocular mobility or extension of the orbicularis oculi muscle 31. However, in cataract surgery, there is an absence of mobility or stretching of the orbicularis oculi muscle.

During the phacoemulsification surgery completed in this study, ocular displacement did not occur, indicating zero stretching of the orbicularis oculi muscle. Hence, this method is a way to circumvent stimulating the oculomotor reflex, which is categorized by compensatory eye movements that position the dorsal ommatidia of the compound eye upwards 32. Pressure stimulation may occur when the phacoemulsification device is used in the intraocular area. Thus, positive pressure to sustain the anterior region of the eye is generated to prevent eye collapse.

Our results advocate monitoring of the HR in patients with cardiac dysfunction during cataract surgery. Our data illustrate the possibility of abrupt reductions in HR. The evaluation of HRV has been described for the diagnosis of physiological dysfunctions 33. However, research related to its use in clinical practice is rare. A previous study from Godoy et al. 34 showed that HRV analysis may provide important results in clinical practice.

In the study by Godoy et al. 34, it was shown that HRV indices can be used to forecast morbidity and mortality in patients undergoing myocardial revascularization surgery. Subjects with reduced complexity of HR dynamics experienced an increase in morbidity and mortality rates.

HRV analysis may be a supplementary approach in clinical practice as a method of monitoring patients undergoing cataract surgery. Moreover, HRV is an important method to explore the autonomic nervous system, which has an important role in the maintenance of homeostasis.

Our study presents some results worth highlighting. We did not collect blood catecholamine levels, which could provide important information for sympathetic nervous system activity. We did not evaluate a control group under placebo conditions for ethical reasons. The ethics committee that approved this research did not permit us to examine the same subjects under control conditions because they required transport from our institution to the necessary hospital. Because our institution has only one bus to transport all patients, the ethics committee determined that the control procedure would cause difficulties to other patients needing transport to the hospital. Nevertheless, the study was performed in a clinical environment; we emphasize the relevance of further laboratory experimental investigations to improve this methodological limitation.

The subjects were evaluated in the morning between 8:00 and 12:00. The cortisol levels are low at 8:00 and high at 12:00 35. Although a previous study suggested a significant influence of cortisol on HRV 36, another investigation was unsuccessful in finding a significant association between cortisol and HRV 37.

We did not evaluate the isolated effect of local intraocular anesthesia on HRV. Previously, it was shown that local anesthesia during ophthalmic surgeries increased the HR and LF/HF ratio 17. Another study showed that alphacaine did not significantly influence HRV in subjects undergoing endodontic treatment due to irreversible pulpitis or pulp necrosis of the upper front teeth 38,39,40. Taken together, the aforementioned studies indicate that intraocular anesthesia is unable to increase the vagal influence on heart rhythm.

The main novel results of our study are as follows: 1) there was a significant difference between factors on the basis of the effect size calculation, strengthening the statistical significance of our results; 2) cardiac disease patients may experience cardiac complications during this ophthalmologic procedure; and 3) the obtained data has high clinical applicability, as this study's conditions were extremely similar to routine clinical situations.

Our data provide relevant information for clinical practice. A retrospective study published in 2009 18 showed that medical teams did not usually consider the oculovagal reflex during surgical procedures. Our results emphasize the importance of monitoring HR dynamics during cataract surgery. Our statistical results indicate that surgeons should pay distinct attention to patients with cardiac dysfunction. Parasympathetic activation during cataract surgery may induce intense bradycardia and lead to cardiac deterioration.

In summary, our study suggests that cataract surgery may increase the parasympathetic control of the HR. This influence may be because of the stimulation of the ophthalmic supraorbital branch of the trigeminal nerve. The lack of a control group reinforces the necessity for further laboratorial studies under experimental conditions. Surgeons should be cautious when performing cataract surgery in patients with a history of cardiac abnormalities to avoid cardiovascular complications during these surgeries.

## AUTHOR CONTRIBUTIONS

Aoki RH, Roque AL, Bezerra IMP, Raimundo RD and Oliveira FR participated in the acquisition of data and manuscript drafting. Almeida-Júnior AD, Barbosa RTA and Oliveira FR participated in the experiments and performed the statistical analysis. Valenti VE performed the data analysis, prepared the figures and tables, and was also responsible for the manuscript drafting and writing. Valenti VE, Abreu LC and Macedo-Júnior H revised the manuscript and participated in the acquisition of data. Valenti VE, Roque AL and Garner DM wrote the manuscript, and helped preparing figures and tables. Abreu LC supervised the study and approved the final version of the manuscript submitted for publication. All authors reviewed and approved the manuscript.

## Figures and Tables

**Table 1:** Comorbidities presented by the included patients.

Disease	Number
Obesity	5 (2 men)
Lumbar hernia	2 (1 man)
Obesity and lumbar hernia	12 (6 men)

**Table 2:** Mean values, followed by their respective standard deviations of the body mass index (BMI), height, mass and age.

Variables	Men	Women
Age (years)	70.2±3.1	73.4±4.1
BMI (kg/m^2^)	69.5±2.82	69±4.7
Caucasian	6	8
Black	2	3

BMI=body mass index; kg=kilogram; m=meter.

**Table 3:** Correlation between age and BMI with resting HRV.

RMSSD	r	*p*
Age	0.004	0.98
BMI	0.01	0.69
SDNN	r	*p*
Age	0.07	0.27
BMI	0.001	0.86
pNN50	r	*p*
Age	0.22	0.06
BMI	0.002	0.94
RRtri	r	*p*
Age	0.07	0.27
BMI	0.01	0.59
TINN	r	*p*
Age	0.17	0.07
BMI	0.01	0.66
SD1	r	*p*
Age	0.15	0.1
BMI	0.01	0.69
SD2	r	*p*
Age	0.06	0.3
BMI	0.003	0.81
LF	r	*p*
Age	0.05	0.34
BMI	0.03	0.45
HF	r	*p*
Age	0.05	0.34
BMI	0.03	0.45
LF/HF	r	*p*
Age	0.006	0.97
BMI	0.02	0.56

Legend**:** pNN50: the percentage of adjacent RR intervals with a difference of duration greater than 50 ms; RMSSD: root-mean square of differences between adjacent normal RR intervals in a time interval; ms: milliseconds; SDNN: average standard deviation of normal RR intervals; SD1: standard deviation of the instantaneous variability of the beat-to beat HR; SD2: standard deviation of long-term continuous RR interval variability; TINN: triangular interpolation of RR interval histogram; RRtri: triangular index; LF: low frequency; HF: high frequency; LF/HF: low frequency/high frequency ratio; BMI: body mass index.
